# Explanatory model of violent behaviours, self-concept and empathy in schoolchildren. Structural equations analysis

**DOI:** 10.1371/journal.pone.0217899

**Published:** 2019-08-16

**Authors:** Manuel Castro-Sánchez, Félix Zurita-Ortega, Gerardo Ruiz-Rico Ruiz, Ramón Chacón-Cuberos

**Affiliations:** 1 Department of Didactics of Musical, Plastic and Corporal Expression, University of Granada, Granada, Spain; 2 Department of Education, University of Almería, Almería, Spain; 3 Department of Research Methods and Diagnosis in Education, University of Granada, Granada, Spain; California State University, Sacramento, UNITED STATES

## Abstract

The increased visibility of bullying cases has led the scientific community to be more interested in analysing the factors affecting these behaviours in order to reduce bullying cases and their negative consequences. The aim of this study was to define and contrast an explanatory model that makes it possible to analyse the relationships between self-concept, empathy and violent behaviours in schoolchildren through structural equation analysis. The sample of this study is made up of 734 schoolchildren from the province of Granada (Spain), both male and female, aged between 10 and 12, and it consists of analysing self-concept (AF-5), empathy levels (TECA) and violent behaviour at schools (ECV). A structural equation model was performed and successfully adjusted (*χ*^2^ = 563.203; *DF* = 59; *p* < 0.001; CFI = 0.943; NFI = 0.937; IFI = 0.943; RMSEA = 0.076). A positive and direct relationship between self-concept and cognitive empathy has been found; manifest aggression is negatively related to self-concept. Similarly, affective empathy has a negative relationship with relational aggression. The main conclusions of this study are that the levels of self-concept and empathy represent protective factors against the development of violent and victimisation behaviours in schoolchildren.

## Introduction

In recent decades, there has been an increase in the visibility of bullying cases, which has led to an increase in the number of reports of such violent behaviours, representing an epidemic in the 21st century [1,2]. Due to greater visibility of the problem and better knowledge of the consequences that bullying can have on younger subjects, sometimes leading to suicide, social awareness of bullying situations has increased, looking for measures to tackle this problem [3–5]. The research developed by Moreno-Jiménez, Rodríguez-Muñoz, Salin and Morante [6] indicated that four out of every five students acknowledged having carried out some type of school violence. Similarly, Rechea [7] highlighted that a 72% of adolescents aged between 12 and 17 years-old had carried out antisocial or criminal behavior in the last year.

Nowadays, the study of school violence has had a great impact on the scientific field, making it a priority subject, as can be seen from the large amount of research currently being carried out on this subject [8,9]. Violence or bullying at school is a way of exercising domination over others by using force or power relationships, ultimately aiming to maintain a supposed superiority over peers [10,11]. Bullying is a type of school violence, since bullying, abuse or harassment, are specific forms of school violence in which the most powerful aggressors cause pain, harass or repress another classmate [12]. There are four traditional forms of bullying: Physical: aggressive behavior directed toward the body or property; Verbal: aggressive behavior directed towards cognition; Social: isolating and excluding behaviors; and Psychological: behaviors that erode self-esteem and cause insecurity and fear [13].

Violence has physical and psychological consequences that are destructive and harmful in spite of it will be a behavior for relationship or a way for conflict resolution between people. The malignancy of the situation is even greater when violence occurs at an early age because adolescents are in a process of primary socialization. Thus, any child or adolescent who is a victim or witness of a violent act not only suffers the immediate and painful consequence, but also internalizes the negative experience through which they themselves learn the process of violent behavior [14].

Due to this problem, countries are implementing various intervention programmes with the aim of reducing the number of violent behaviours taking place at a school age, and more specifically in the school environment, focusing on making pupils, families and teachers aware of all aspects related to bullying [15,16]. For these reasons, it is essential to identify and control the risk factors that influence the development of violent behaviour in schoolchildren, since at these ages behavioural patterns are created that, if strengthened, will be maintained during adolescence and adulthood [17,18].

One of the main psychological factors in student development is self-concept, which is defined as the subject’s perceptions of himself or herself in different areas or levels, such as physical, emotional, academic, social and family [19]. Several authors point out the importance of self-concept as one of the fundamental factors involved in the configuration of the subject’s personality, affecting the well-being of the individual [20]. This factor is related to the development of violent behaviours, based on various research that associates low levels of self-concept with a higher likelihood of being aggressors or undergoing victimisation processes [21].

Several studies have analysed the relationship between self-concept and violent behaviours, and they have shown that this psychological factor has an effect on victimisation, since subjects with low levels of self-concept are more likely to undergo situations of harassment, affecting them greatly, which may in turn lead to the development of aggressive behaviours; while subjects with high levels of self-concept undergo less victimisation and, therefore, develop less aggressiveness [22,23].

Another psychological factor related to the development of violent behaviours is empathy, which is defined as the ability to understand other people’s feelings and emotions, or the ability to put oneself in other people’s place [24]. Empathy is made up of two large dimensions, cognitive empathy and affective empathy, the first refers to the ability to communicate, maintain effective social relationships, tolerate others and recognise and understand emotions and impressions in an optimal way; and the second, defined as the ability to understand and share other people’s emotions, both positive and negative [25].

Several studies have analysed the relationships between the levels of empathy and the development of violent, aggressive and victimisation behaviours in different populations, showing that lower levels of empathy increase the development of manifest and relational aggressiveness, while high levels of empathy decrease the emergence of violent behaviours, due to the fact that when the individual is able to identify other people’s feelings, he or she avoids causing any harm [26,27]. Regarding the association between self-concept and empathy, a positive relationship between both psychological factors has been demonstrated. In fact, it is observed that when empathy increases, so does self-concept due to a better understanding and internalization of the psychosocial reality of the other and its influence on each individual’s own [28].

Therefore, due to the importance of self-concept and empathy in the development of violent behaviours, a theoretical explanatory model of structural equations has been developed. The aim of this model is to define and contrast an explanatory model that makes it possible to analyse the relationships between self-concept, empathy and violent behaviours in schoolchildren through structural equation analysis.

## Methods

### Design and participants

This research work uses a descriptive cross-sectional design, analysing a sample consisting of 734 schoolchildren male and female (45.2% boys and 54.8% girls), aged between 10 and 12 (M = 10.88 years; DT = 0.69), belonging to third cycle (fifth and sixth year) of Primary Education in the city of Granada (Spain). The sample has been selected through a convenience sampling process, considering as selection criteria: (a) to take the fifth or sixth year of Compulsory Primary Education in the city of Granada; (b) to obtain the informed and written consent from parents or legal guardians. The proposed exclusion criteria was: (a) not suffer from any type of pathology that could prevent the correct application of instruments and scales. In addition, it is important to highlight that due to a convenience sampling has been carried out, the results obtained in the present research should be interpreted with caution. This sample comes from eleven education centres in the city of Granada. All education centres in the city that agreed to collaborate in the research on a voluntary basis were asked to participate. Finally, it should be mentioned that the researchers were present during data collection so that the process could be developed in an optimal way, avoiding subjects not being repeated, in order to avoid data duplication.

### Instruments

The following variables were collected:

1. Self-concept Form-5 Questionnaire (AF-5, in Spanish), developed by García et al. [29] and based on the theoretical model of Shavelson et al. [30]. It consists of 30 items that are scored using a 5-option Likert scale, where 1 is ‘Never’ and 5 is ‘Always’ (e.g.: *I do well school work*). Self-concept is grouped into five dimensions according to this instrument, which are the following: academic self-concept (items 1, 6, 11, 16, 21 and 26), social self-concept (items 2, 7, 12, 17, 22 and 27), emotional self-concept (items 3, 8, 13, 18, 23 and 28), family self-concept (items 4, 9, 14, 19, 24 and 29), and physical self-concept (items 5, 10, 15, 20, 25 and 30). The study carried out by García et al. [29] established a reliability (determined by Cronbach’s alpha coefficient) of α = 0.810, a value similar to that identified in this research work (α = 0.832).
2. Cognitive and Affective Empathy Test (TECA, in Spanish), developed by López-Pérez et al. [31], whose main objective is to analyse the empathic ability of the subject from a double, cognitive and affective perspective. The questionnaire consists of 33 items that are scored using a 5-option Likert scale, where 1 is ‘Strongly disagree’ and 5 is ‘Strongly agree’ (e.g.: *I feel good if others have fun*). The instrument introduces a tetra-factorial model, consisting of two large dimensions, cognitive empathy and affective empathy. These are in turn made up of two factors each, perspective taking (subject’s ability to communicate, tolerate others and maintain social relationships; items 6, 11, 15, 17, 20, 26, 29 and 32) and emotional comprehension (ability to recognise and understand other people’s emotions and impressions; items 1, 7, 10, 13, 14, 24, 27, 31 and 33), which make up cognitive empathy, and empathic stress (ability to understand and share other’s people negative emotions; items 3, 5, 8, 12, 18, 23, 28 and 30) and empathic joy (ability to understand and share other people’s positive emotions; items 2, 4, 9, 16, 19, 21, 22 and 25), which make up affective empathy. The study carried out by López-Pérez et al. [31] established a reliability of α = 0.820, a value similar to that found in this research work (α = 0.803).
3. Violent Behavior at School (ECV, in Spanish), divided into two categories, Manifest Aggression and Relational or Indirect Aggression, further subdivided into three sub-scales (Pure, Reactive or Instrumental) and analysed through the Scale of Violent Behaviour at School. This scale is proposed in its original version by Little et al. [32], adapted by the Lisis Group [33], and used in studies with similar characteristics, including Cava et al. [34]; Musitu et al. [35] or Jiménez et al. [36]. It consists of a 25-item Likert scale ranging from values 1 (never) to 4 (always) (e.g.: *I am a person who fights with others*), which, once scored, result in two types of violent behaviour: Manifest or Direct Aggression (arising from a face-to-face encounter in which the aggressor can be identified by the victim) or Relational or Indirect Aggression (considered when the aggressor remains anonymous). Violent overt behaviors refer to situations in which there is some type of physical or verbal aggression, such as hitting or insulting a partner directly. The type of relational violence refers to the behaviors related to the exclusion of a member of the peer group, performing these behaviors indirectly or without a direct contact with the victim. In addition, there are three subtypes of violence: the pure -carried out as spontaneous behavior-, the instrumental -when aggressor seeks to obtain a benefit derived from that aggression-, and reactive -made as a reaction to a certain previous behavior-. They show a Cronbach’s Alpha reliability coefficient of α = 0.856 for items measuring manifest aggressiveness and α = 0.742 for relational aggressiveness issues, very similar to α = 0.880 and α = 0.810, for both sub-scales from Musitu et al. [35].

### Procedure

Through the Faculty of Education of the University of Granada, and in contact with the Education Office of the Regional Government of Andalusia, education centres from the city of Granada were requested to collaborate, inviting them to participate, by using a convenience sampling process. Each education centre’s management was informed of the nature of the research, requesting student collaboration. Once they agreed to participate in the research, an authorisation model was provided to those legally responsible for schoolchildren, requesting their informed consent, since the participants of this research were minors. Finally, the informed consent of the legal guardians was successfully obtained in writing for all the subjects who participated in the present study.

It should be noted that the anonymity of the participants was ensured at all times, and they were informed that the data would only be used for scientific purposes. To that end, the researchers were present during the data collection so that the process could be carried out in an optimal way, solving any doubts related to the completion of the questionnaire. Finally, the management team, the teachers involved and the students were thanked for their participation and collaboration in the study, and they were informed that a report with the data obtained in their education centre will be sent in the future, while maintaining the confidentiality of the students and for information purposes only.

As for questionnaires, 52 of them were rejected because they were incorrectly completed or they presented questions that had not been completed. This research work has followed the guidelines set out in the Declaration of Helsinki (World Medical Association, 2008), concerning research projects, in addition to the national legislation for clinical trials (Royal Decree 223/2004, of 6th February), biomedical research (Act 14/2007, of 3rd July) and for confidentiality of the participants (Act 15/1999, of 13th December). Permission was obtained from the University of Granada´s Ethics Committee for Research (641/CEIH/2018).

### Data analysis

The IBM SPSS statistical software version 22.0 for Windows was used in order to perform basic descriptive analyses. The IBM AMOS 23 programme was used to analyse the relationships between the constructs involved in the structural model. Once the theoretical model has been developed, a path analysis is performed based on the relationships of the matrix from a structural equation analysis.

**Fig 1:**
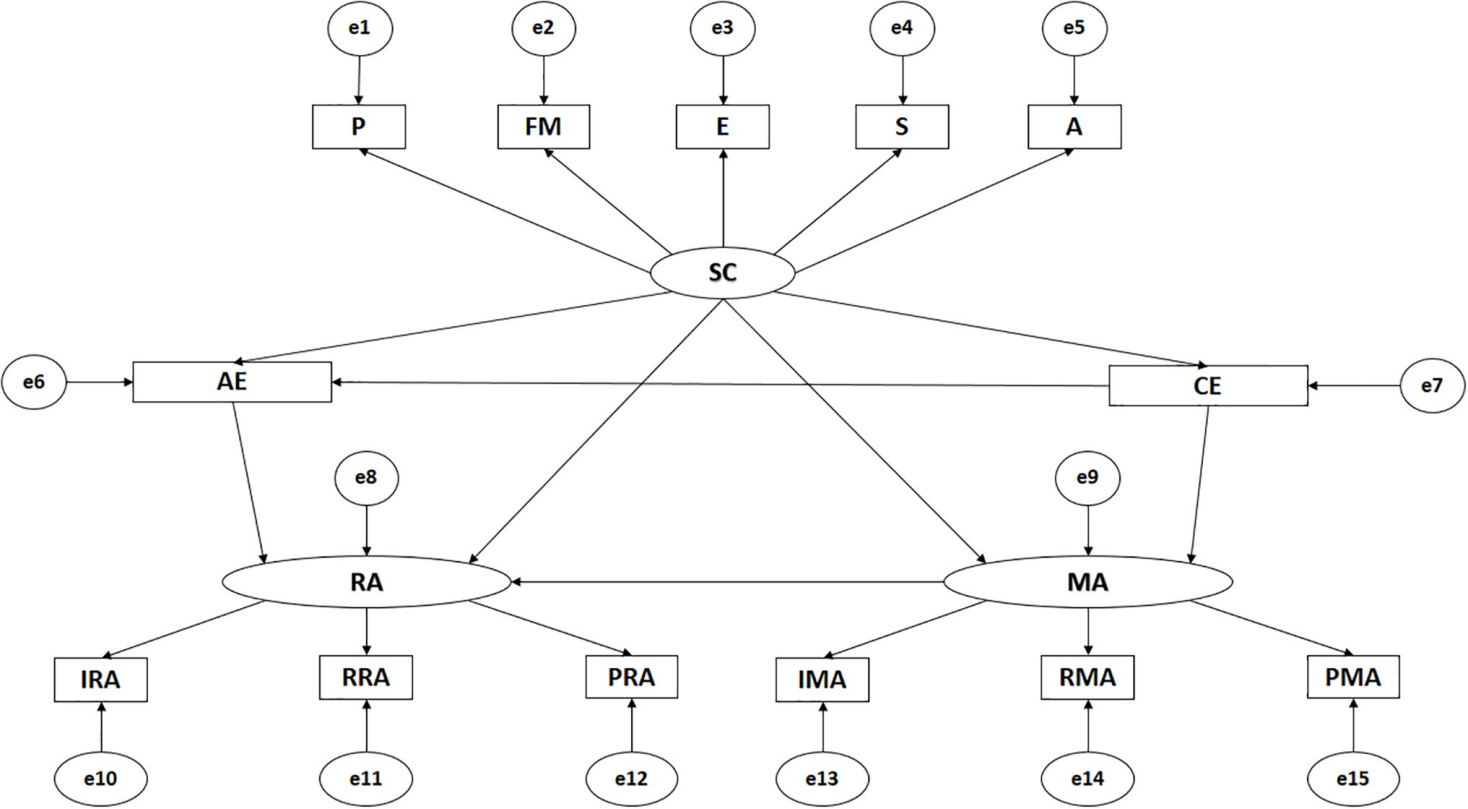
Model theorise: Self-concept, empathy and violent behaviour. *Note 1*: SC, Self-concept; P, Physical self-concept; FM, Family self-concept; E, Emotional self-concept; S, Social self-concept; A, Academic self-concept; AE, Affective Empathy; CE, Cognitive Empathy; MA, Manifest Aggression; RA; Relational Aggression; IMA, Instrumental Manifest Aggression; RMA, Reactive Manifest Aggression; PMA, Pure Manifest Aggression; IRA, Instrumental Relational Aggression; RRA, Reactive Relational Aggression; PRA, Pure Relational Aggression.

The path analysis is made up of thirteen observable variables and thirteen latent variables to determine the indicators (Fig 1). In these models, causal explanations of latent variables are provided based on the relationships observed between indicators, taking into account the reliability of the measurements. Likewise, measurement errors are included in the observable variables so that they are directly controlled. Unidirectional arrows are lines of influence between latent and observable variables, and these are interpreted as multivariate regression coefficients. Bidirectional arrows show the relationship between latent variables, also representing regression coefficients.

Self-concept (SC) acts as an exogenous variable and is inferred from five indicators. The indicators for self-concept are P (Physical), FM (Family), E (Emotional), S (Social) and A (Academic). Cognitive empathy (CE) acts as an endogenous variable, receiving the effect of self-concept (SC). Affective empathy (AE) acts as an endogenous variable, receiving the effect of self-concept (SC) and cognitive empathy (CE). Manifest aggression (MA) is inferred from three indicators and acts as an endogenous variable, receiving the effect of self-concept (SC) and cognitive empathy (CE). The indicators for manifest aggression (MA) are IMA (Instrumental Manifest Aggression), RMA (Reactive Manifest Aggression) and PMA (Pure Manifest Aggression). Similarly, the relational aggression (RA) is inferred from three indicators and acts as an endogenous variable, receiving the effect of self-concept (SC) and affective empathy (AE) and manifest aggression (MA). The indicators for relational aggression (RA) are IRA (Instrumental Relational Aggression), RRA (Reactive Relational Aggression) and PRA (Pure Relational Aggression).

The fit of the model was checked in order to verify its compatibility and the empirical information obtained. The reliability of the fit was based on Marsh’s goodness-of-fit criteria [37]. In the case of Chi-square test, the non-significant values associated with p indicate a good fit of the model. The value of the Comparative Fit Index (CFI) will be acceptable with values greater than 0.90, and excellent, for values greater than 0.95. The Normalised Fit Index (NFI) should be greater than 0.90. The value of the Incremental Fit Index (IFI) will be acceptable with values greater than 0.90, and excellent, for values greater than 0.95. Finally, the Root Mean Square Error of Approximation (RMSEA) will be excellent if less than 0.05, and acceptable, if less than 0.08.

## Results

Table 1 shows the descriptive data of the self-concept, empathy and violent behavior of the sample, observing both the total scores and the differences by sex.

**Table 1:** Descriptive analysis of AF-5, TECA and ECV.

		Total	Sex		P-value
		(n = 734)	Boys (n = 332)	Girls (n = 402)	
**Self-Concept**	Academic Self-Concept	3.85 ± 0.81	3.71 ± 0.84	3.97 ± 0.77	0.000***
	Social Self-Concept	3.88 ± 0.77	3.82 ± 0.81	3.93 ± 0.74	0.008***
	Emotional Self-Concept	3.05 ± 0.90	3.04 ± 0.85	3.06 ± 0.94	0.695
	Family Self-Concept	4.12 ± 0.88	4.05 ± 0.91	4.18 ± 0.86	0.003***
	Physical Self-Concept	3.66 ± 0.78	3.64 ± 0.78	3.68 ± 0.77	0.348
**Empathy**	**Cognitive Empathy**	3.30 ± 0.58	3.24 ± 0.61	3.36 ± 0.55	0.000***
	Perspective Adoption	3.26 ± 0.65	3.19 ± 0.67	3.33 ± 0.62	0.000***
	Emotional Understanding	3.34 ± 0.66	3.28 ± 0.67	3.39 ± 0.64	0.002***
	**Affective Empathy**	2.93 ± 0.54	2.91 ± 0.49	2.95 ± 0.58	0.194
	Empathic Stress	2.67 ± 0.67	2.68 ± 0.64	2.66 ± 0.69	0.604
	Empathic Joy	3.19 ± 0.73	3.14 ± 0.60	3.23 ± 0.81	0.016*
**Violent Behaviour**	**Manifest Aggression**	1.31 ± 0.44	1.35 ± 0.44	1.27 ± 0.45	0.000***
	Instrumental Manifest Aggression	1.23 ± 0.48	1.25 ± 0.48	1.22 ± 0.49	0.170
	Reactive Manifest Aggression	1.39 ± 0.59	1.49 ± 0.62	1.31 ± 0.55	0.000***
	Pure Manifest Aggression	1.32 ± 0.45	1.35 ± 0.46	1.29 ± 0.43	0.018*
	**Relational Aggression**	1.39 ± 0.46	1.40 ± 0.46	1.39 ± 0.46	0.670
	Instrumental Relational Aggression	1.26 ± 0.50	1.28 ± 0.52	1.25 ± 0.49	0.369
	Reactive Relational Aggression	1.58 ± 0.59	1.60 ± 0.61	1.57 ± 0.57	0.275
	Pure Relational Aggression	1.34 ± 0.51	1.33 ± 0.48	1.35 ± 0.54	0.325

Note 1:

*** Statistically significant relationship between variables at 0.005 level

* Statistically significant relationship between variables at 0.05 level.

Taking into account each dimension of self-concept, the most valued dimension was family (4.12 ± 0.88), followed by social (3.88 ± 0.77), academic (3.85 ± 0.81) and physical dimensions (3.66 ± 0.78), being the emotional dimension the worst valued (3.05 ± 0.90). Regarding the differences by sex, statistical association was found (p < 0.005) in the academic, social and family dimensions, with girls showing a higher self-concept in these dimensions.

Regarding the levels of empathy, it has been found that cognitive empathy (3.30 ± 0.58), and its dimensions adopting perspective (3.26 ± 0.65) and emotional comprehension (3.34 ± 0.66) obtained higher scores than the affective dimension (2.93 ± 0.54) and its corresponding two categories, empathic stress (2.67 ± 0.67) and empathic joy (3.19 ± 0.73). Considering the differences by sex, statistical association was found (p < 0.005) in the cognitive empathy, and its dimensions adopting perspective and emotional comprehension, showing how females obtained higher values. In the case of affective empathy, there was only statistical association (p < 0.05) in the case of empathic joy, showing higher values for boys.

Finally, when the violent behaviors are analysed, it was observed how the relational aggressiveness (1.39 ± 0.46) presented higher scores than the manifest aggressiveness (1.31 ± 0.44), as it happened with their respective dimensions. Analysing the differences between sexes, the statistical non-association (p ≥ 0.05) in the relational aggressiveness and its dimensions was verified. However, in the case of overt aggression and in the reactive and pure dimensions, the boys had higher scores than the girls.

The proposed structural equation model reveals a good fit in all assessment indices. The Chi-square test shows a significant value of p (χ2 = 563.203; DF = 59; p < 0.001). However, this index cannot be interpreted in a standardised way, in addition to the problem of its sensitivity to sample size [37]. Thus, other normalised fit indices that are less sensitive to sample size are used. The Comparative Fit Index (CFI) obtains a value of 0.943, which is acceptable. The Normalised Fit Index (NFI) resulted in a value of 0.937, and the Incremental Fit Index (IFI), a value of 0.943, both of which are acceptable. The Root Mean Square Error of Approximation (RMSEA) obtains an acceptable value of 0.076.

**Fig 2:**
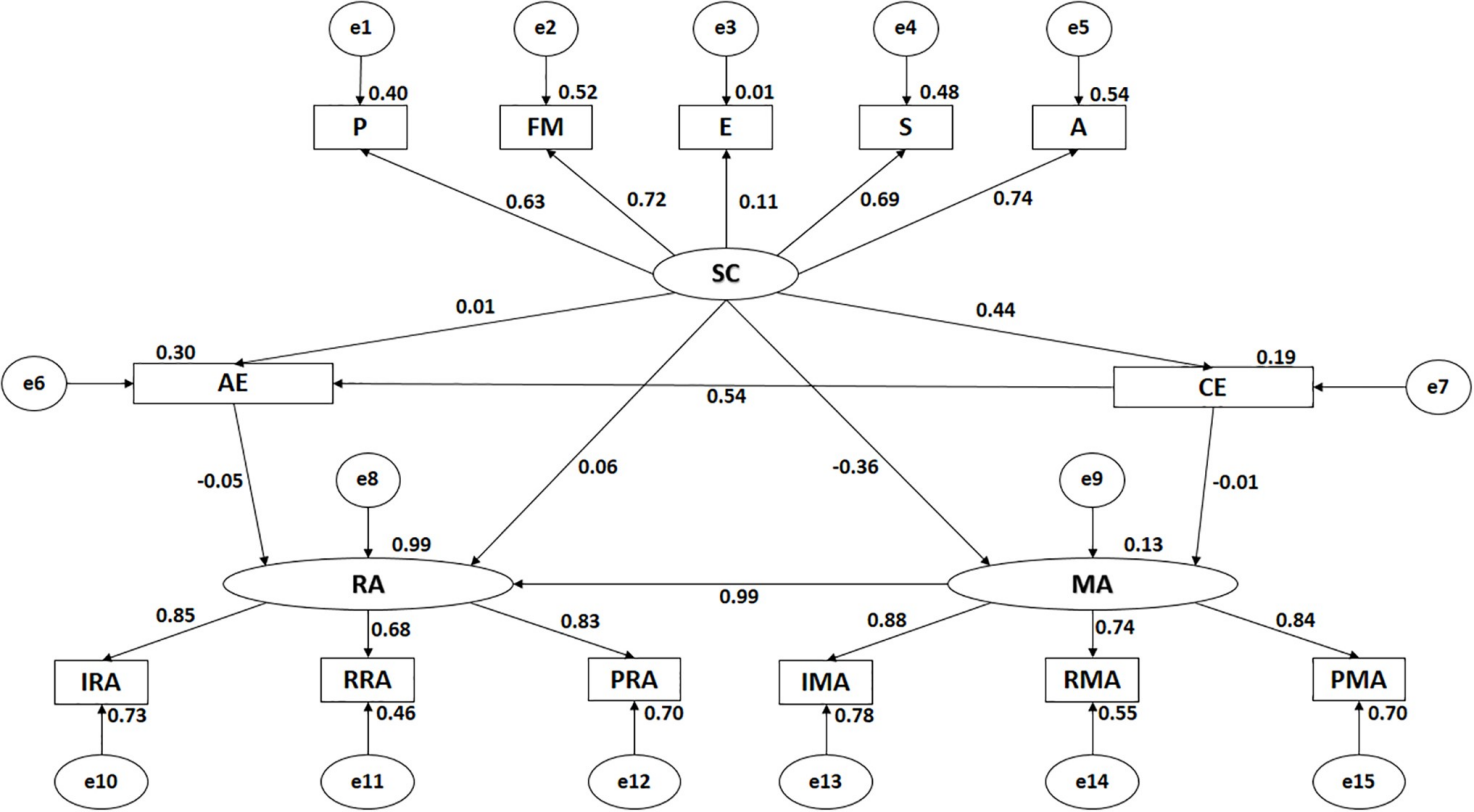
Structural equation model. *Note 1*: SC, Self-concept; P, Physical self-concept; FM, Family self-concept; E, Emotional self-concept; S, Social self-concept; A, Academic self-concept; AE, Affective Empathy; CE, Cognitive Empathy; MA, Manifest Aggression; RA; Relational Aggression; IMA, Instrumental Manifest Aggression; RMA, Reactive Manifest Aggression; PMA, Pure Manifest Aggression; IRA, Instrumental Relational Aggression; RRA, Reactive Relational Aggression; PRA, Pure Relational Aggression.

The estimated values of the structural model parameters are shown in Fig 2 and Table 2. They must be of an appropriate magnitude and the effects must be significantly different from zero. Similarly, inappropriate estimates, such as negative variances, should not be obtained.

**Table 2:** Structural model values.

Relationships between variables			P.R.U.				P.E.R.
			Estimates	S.E.	C.R.	P	Estimates
CE	←	SC	0.522	0.037	14.114	***	0.439
MA	←	SC	-0.277	0.028	-9.883	***	-0.362
AE	←	SC	0.012	0.031	0.375	0.708	0.010
MA	←	CE	-0.006	0.019	-0.312	0.755	-0.009
AE	←	CE	0.506	0.023	21.767	***	0.545
RA	←	SC	0.056	0.017	3.350	***	0.064
RA	←	MA	1.159	0.032	36.176	***	0.992
RA	←	AE	-0.039	0.012	-3.251	***	-0.049
P	←	SC	1.000	-	-	-	0.631
FM	←	SC	1.302	0.063	20.811	***	0.723
E	←	SC	0.205	0.054	3.810	***	0.111
S	←	SC	1.096	0.054	20.285	***	0.693
A	←	SC	1.218	0.058	21.010	***	0.735
PMA	←	MA	1.000	-	-	-	0.837
RMA	←	MA	1.167	0.036	32.726	***	0.743
IMA	←	MA	1.145	0.027	42.464	***	0.882
PRA	←	RA	1.000	-	-	-	0.834
RRA	←	RA	0.936	0.033	28.720	***	0.678
IRA	←	RA	1.009	0.025	39.971	***	0.854

*Note 1*: SC, Self-concept; P, Physical self-concept; FM, Family self-concept; E, Emotional self-concept; S, Social self-concept; A, Academic self-concept; AE, Affective Empathy; CE, Cognitive Empathy; MA, Manifest Aggression; RA; Relational Aggression; IMA, Instrumental Manifest Aggression; RMA, Reactive Manifest Aggression; PMA, Pure Manifest Aggression; IRA, Instrumental Relational Aggression; RRA, Reactive Relational Aggression; PRA, Pure Relational Aggression.

*Note 2*: Unstandardized Regression Weights (P.R.U., in Spanish); Standardised Regression Weights (P.E.R., in Spanish); S.E., Standard Error; C.R., Critical Ratio.

*Note 3*:

*** Statistically significant relationship between variables at 0.005 level

* Statistically significant relationship between variables at 0.05 level.

Statistically significant relationships are observed at p < 0.005 level between all Self-concept dimensions, which are positive and direct. Likewise, statistically significant relationships are observed at p < 0.005 level between violent behaviours (manifest aggression and relational aggression) and their indicators, with all the associations being positive and direct.

By analysing the influence of the indicators in each latent variable, it could be noted that all of them had statistically significant differences at p < 0.005 level, with all the relationships being positive and direct. In the case of Self-concept, the Academic dimension is the indicator that shows the highest correlation coefficient (r = 0.735), followed by Family self-concept (r = 0.723), Social self-concept (r = 0.693) and Physical self-concept (r = 0.631), being Emotional self-concept the one that shows the lowest correlation coefficient (r = 0.111). For Manifest Aggression, the greatest association is found in Instrumental Manifest Aggression (r = 0.882), followed by Pure Manifest Aggression (r = 0.837) and Reactive Manifest Aggression (r = 0.743). For Relational Aggression, the greatest association is found in Instrumental Relational Aggression (r = 0.854), followed by Pure Relational Aggression (r = 0.834) and Reactive Relational Aggression (r = 0.678).

Similarly, significant associations (p < 0.005) are observed in the relationship between Self-concept and Cognitive Empathy–which is positive and direct (r = 0.439). However, there is no association between Self-concept and Affective Empathy (p = 0.708). A negative and indirect association (r = -0.362) is observed between Self-concept and Manifest Aggression at p = 0.005 level, also being found a statistical association (p < 0.005) between Self-concept and Relational Aggression–which is positive and direct (r = 0.064), although the strength of correlation is weak.

Cognitive Empathy and Affective Empathy show a positive and direct relationship at p < 0.005 level (r = 0.545), with a moderate correlation strength, and no association was found in the relationship between Cognitive Empathy and Manifest Aggression (p = 0.755). In the case of Affective Empathy, this is negatively and indirectly related to Relational Aggression, with statistically significant differences at p < 0.005 level (r = -0.049) and low correlation strength. Finally, there is a positive and direct relationship at p < 0.005 level between Relational Aggression and Manifest Aggression (r = 0.992), with high correlation strength.

## Discussion

Reviewing the scientific literature associated with the factors analysed in this research, we find an inverse association between violent behaviors and levels of empathy, showing how subjects with higher levels of empathy are more reluctant to perpetrate and observe the violent behavior [38–39]. Regarding self-concept and violent behavior, several international studies show an inverse association between these factors, finding that individuals with greater self-concept are less likely to develop violent behavior [40]. Finally, a positive and direct association has been found between the levels of empathy of the pre-adolescents and their self-concept [35]. Due to these reasons, this research presents a model of structural equations that explains the connection between the psychosocial factors mentioned, based on the scientific literature analysed.

This study performs a structural equation analysis in order to contrast the associations between violent behaviours, self-concept and empathy in a sample of schoolchildren. The path model developed obtains acceptable fit indices, forming a valid explanatory model that makes it possible to explain the relationships between the factors of violent behaviours, self-concept and empathy, as several studies have done at a national and international level in recent years [41–47].

When the influence of self-concept indicators is analysed, it is noted that the academic, family and social dimensions have the greatest influence on the overall self-concept of schoolchildren, with the physical and emotional dimensions having the least influence on the overall construct. This can be explained by the age of the subjects in the study sample, since at school age the subjects attach great importance to their family relationships, the marks they obtain and their social relationships [48,49]. However, when subjects get older (i.e. during adolescence), physical and emotional self-concept is particularly important, turning to be paramount, because this period represents one of the most convulsive moments of the person at a psychological level, as well as the perception of his or her own physical appearance becomes particularly relevant since he or she becomes interested in the opposite sex at this age, seeking to be deemed attractive and to meet the beauty canons [50–52].

In terms of violent behaviours, it is noted that in both relational aggression and manifest aggression, the type of aggression that has the greatest influence or that is most commonly used is instrumental aggression. At these ages, schoolchildren use violent behaviours, whether manifest or relational, in order to achieve a certain goal, intending to benefit from the victim or peer group [53,54]. As subjects get older, violent behaviours of pure and reactive nature take place to a greater extent, a period that usually coincides with the start of adolescence [55].

In analysing the relationships between the levels of self-concept and empathy in the schoolchildren studied, a positive and direct relationship between self-concept and cognitive empathy was found. This means that schoolchildren with better self-concept have greater empathic abilities in their cognitive dimension, so that they are more effective in communicating, tolerating others and maintaining social relationships; as well as they have better abilities to recognise and understand other people’s emotions and impressions [56].

There is a negative relationship between self-concept and manifest aggression in the schoolchildren analysed, and this data agrees with that found in all studies consulted [57–59]. This data can be explained by the fact that there is a profile of subjects who engage in violence because of their low level of self-concept, using these violent behaviours as a means of concealing their perception of themselves [60]. A positive relationship has also been found between self-concept and relational aggression, which, although significant, has a very weak correlation strength, so that it may be due to the characteristics of the sample, rather than to a generally applicable result, with inconsistent data being found in scientific literature [61]. This may be explained because subjects with greater self-concept feel more capable of manipulating others and engaging in this type of violence that is subtler than manifest violence.

On the other hand, there is a negative relationship between affective empathy and relational aggression. Schoolchildren with higher levels of affective empathy are less likely to engage in relational violence. This may be explained because, when the subject is able to put himself or herself in his or her peers’ shoes, he or she is aware of the harm that can be caused by engaging in a type of violence that isolates or keeps a colleague away from the peer group, so that he or she will be less likely to engage in this type of aggression [62,63].

Affective empathy and cognitive empathy are positively related, so when one increases, the other does too, concluding that schoolchildren with higher levels of cognitive empathy show better levels of affective empathy and vice versa. The two dimensions of empathy are related, since they have similar abilities, such as putting oneself in other people’s shoes, and identifying and understanding other people’s feelings and emotions [64,65]. The same is true in the case of violent behaviours, where there is a direct and positive relationship between relational aggression and manifest aggression. Schoolchildren who use relational violence are more likely to exercise manifest violence, or vice versa, due to the fact that one type of aggressiveness complements the other [66,67].

This research work shows a number of limitations, since it is a descriptive cross-sectional study, and it does not make it possible to establish cause and effect relationships between variables due to the statistical technique used. Likewise, it is necessary to indicate that the sample analysed is not very high, and this may affect the generalization of the findings obtained. In this sense, the data should be interpreted with caution since it is not a representative sample for the Spanish context. The associations found in this study point to the need for the development of intervention programmes at schools in order to increase the levels of self-concept and empathy of students, because this will reduce the violent behaviours, i.e. bullying, that takes place in education centres. It would also be interesting in future research work if several psychological factors that can have an effect on the development of violent behaviours, such as life satisfaction, motivational factors or factors related to attitudes towards authority, could be added to the theoretical model.

## Conclusions

The main conclusions of this research work are that the indicator with the greatest influence on self-concept is its academic dimension, followed by family, social, physical and emotional. With regard to violent behaviours, the type of aggression, both manifest and relational, that has the greatest influence is instrumental aggression, followed by pure and reactive aggressions.

There is a positive and direct relationship between self-concept and cognitive empathy, but this relationship was not found with affective empathy. As for manifest aggression, there is a negative and indirect association with self-concept, the higher the level of self-concept, the lower the levels of manifest aggressiveness; however, the relationship of self-concept and relational aggression is positive and direct, although it has a very weak correlation strength.

Cognitive and affective empathies have a positive relationship, whereas cognitive empathy and manifest aggression are not related. Affective empathy is negatively related to relational aggression, and thus subjects with higher levels of this type of empathy engage in less relational aggressions. Finally, a positive relationship between relational aggression and manifest aggression is found, meaning that when one increases, the other does too.

As a final conclusion of the present research, the negative association between self-concept and violent behavior in children is highlighted, this being the psychological factor that must be worked from an early age to prevent violent behavior derived from a negative internalization of the self and a low self-esteem.

In this way, several practical implications can be developed focused on the development of intervention programs in educational centres in order to reduce violent behavior among preadolescents. Due to the associations found, it can be indicated that the intervention programs should promote the improvement of self-concept, being essential the collective construction of a positive perception of its different dimensions. It would also be essential to work on empathy as a social skill, which will help potential aggressors to put themselves in the place of the victim, decreasing the prevalence of these violent behaviors. Even so, there is a need to analyse other constructs of interest, such as self-esteem, stress or anxiety. This will allow us to know what factors can be linked to this problem and thus be able to carry out effective intervention programs for the treatment and prevention of this problem.
